# Supplementary figures and images for: Low-glutathione mutants are impaired in growth but do not show an increased sensitivity to moderate water deficit
Source: PLoS One. 2019 Oct 18;14(10):e0220589. doi: 10.1371/journal.pone.0220589 (PMC6799929; doi:10.1371/journal.pone.0220589)

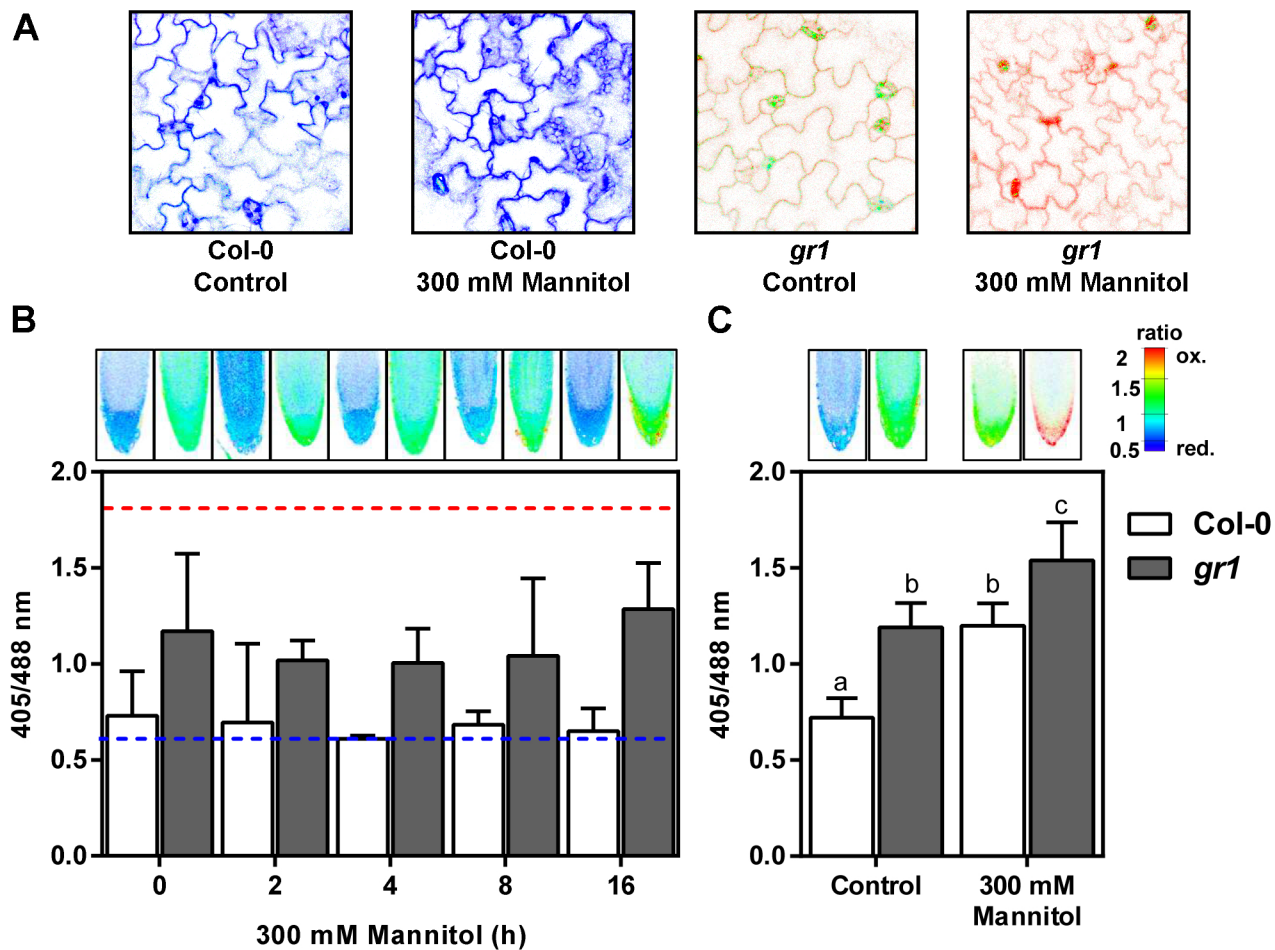

**S1 Fig.**

Supplement: S1 Fig — (A) Cotyledons of 9-day-old seedlings transferred to 300 mM Mannitol on day 5 after germination. (B) 5-day-old seedlings transferred to 300 mM mannitol. The dashed horizontal lines indicate the ratio values resulting from treatment with 10 mM DTT for full reduction (blue) and 25 mM H2O2 for full oxidation (red). (C) 5-day-old seedlings germinated and continuously grown on 300 mM mannitol. All values are means ± SD (n ≥ 10). Letters indicate significant differences (One-way ANOVA with Tukey's multiple comparisons test; p ≤ 0.05). (PDF) [file pone.0220589.s001.pdf]

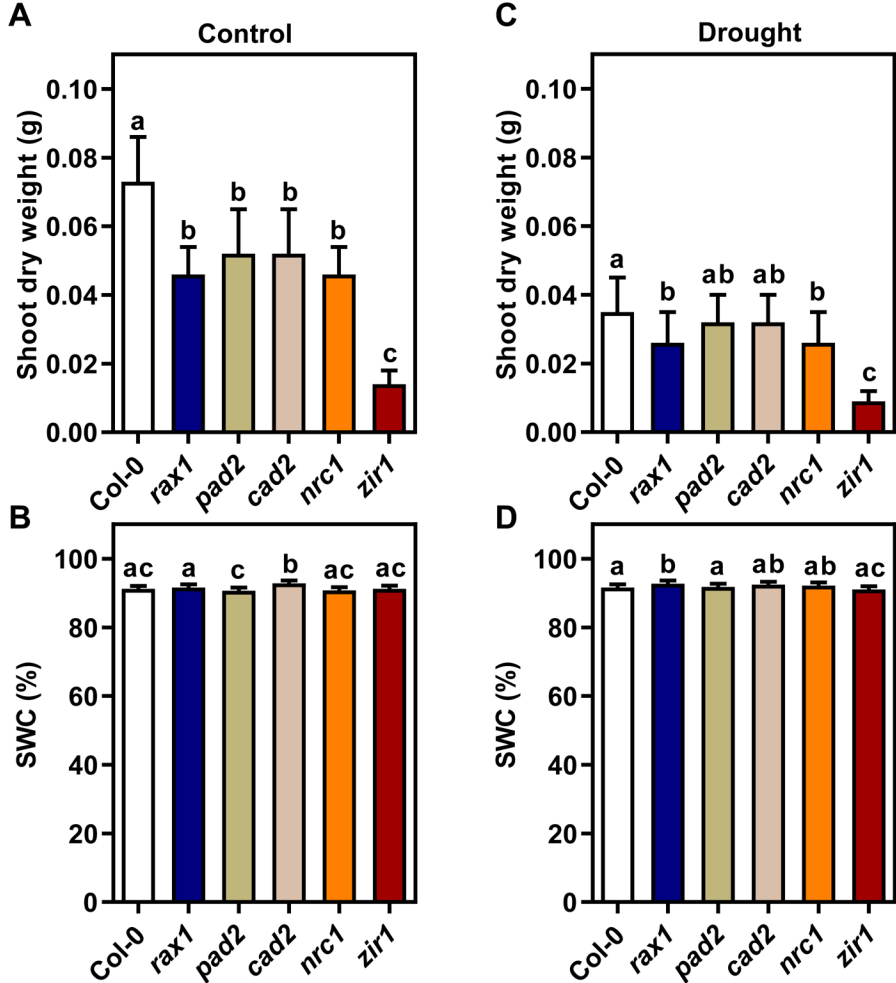

**S2 Fig.**

Supplement: S2 Fig — (A,C) Shoot dry weight for well-watered control plants (A) and drought-stressed plants (C). (B,D) shoot water content under control (B) and drought conditions (D). Values are means ± SD (n ≥ 10). Letters in each graph indicate significant differences (One-way ANOVA with Tukey's multiple comparisons test; p < 0.05). (PDF) [file pone.0220589.s002.pdf]

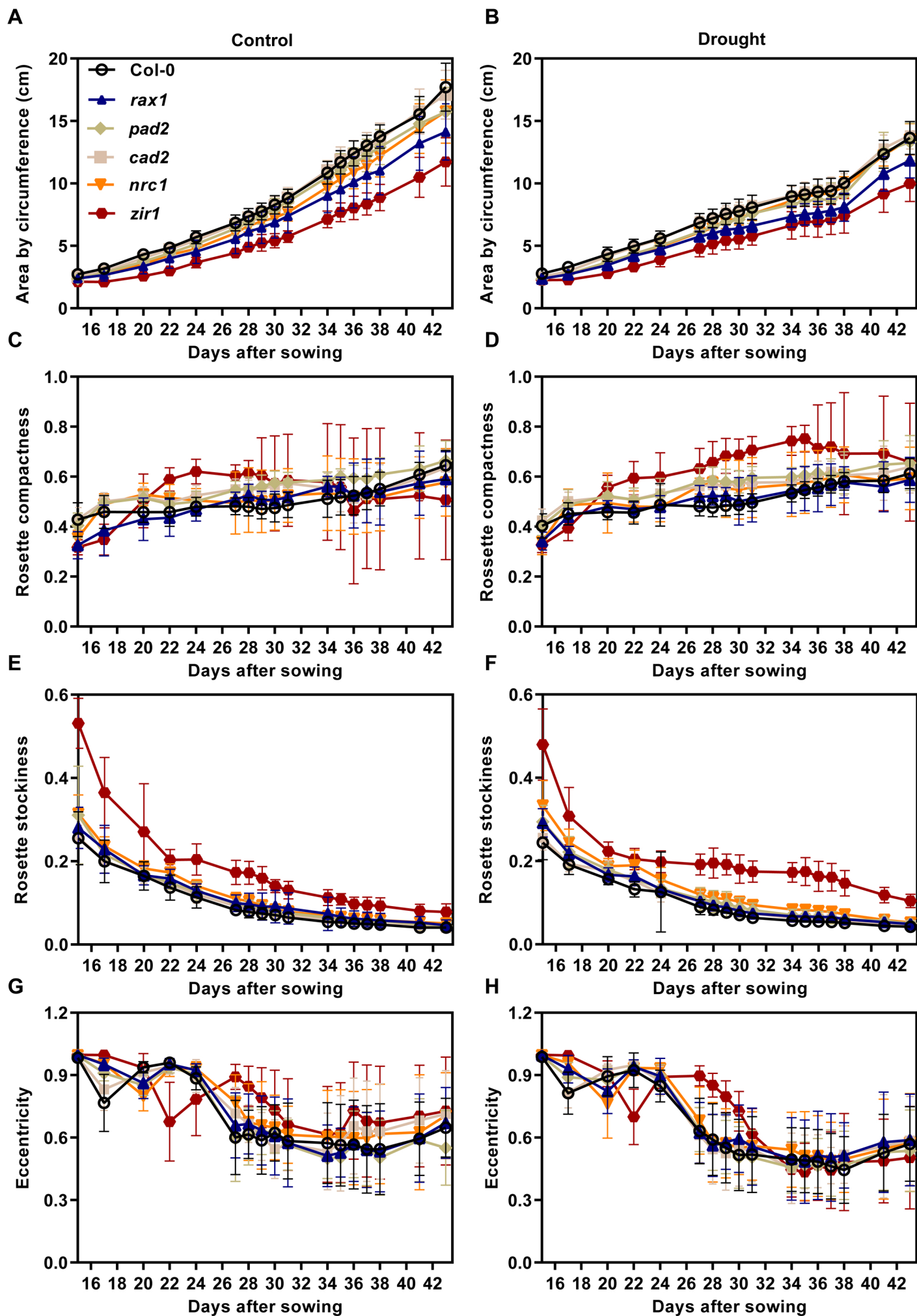

S3 Fig.

Supplement: S3 Fig — (PDF) [file pone.0220589.s003.pdf]
